# Wnt/β-catenin and LIF–Stat3 signaling pathways converge on Sp5 to promote mouse embryonic stem cell self-renewal

**DOI:** 10.1242/jcs.177675

**Published:** 2016-01-15

**Authors:** Shoudong Ye, Dongming Zhang, Fei Cheng, Daniel Wilson, Jeffrey Mackay, Kan He, Qian Ban, Feng Lv, Saifei Huang, Dahai Liu, Qi-Long Ying

**Affiliations:** 1 Center for Stem Cell and Translational Medicine, School of Life Sciences, Anhui University, Hefei 230601, People's Republic of China; 2 Eli and Edythe Broad Center for Regenerative Medicine and Stem Cell Research at USC, Department of Stem Cell Biology and Regenerative Medicine, Keck School of Medicine, University of Southern California, Los Angeles, CA 90033, USA

**Keywords:** Embryonic stem cell, Self-renewal, β-catenin, Stat3, Sp5

## Abstract

Activation of leukemia inhibitor factor (LIF)–Stat3 or Wnt/β-catenin signaling promotes mouse embryonic stem cell (mESC) self-renewal. A myriad of downstream targets have been identified in the individual signal pathways, but their common targets remain largely elusive. In this study, we found that the LIF–Stat3 and Wnt/β-catenin signaling pathways converge on Sp5 to promote mESC self-renewal. Forced *Sp5* expression can reproduce partial effects of Wnt/β-catenin signaling but mimics most features of LIF–Stat3 signaling to maintain undifferentiated mESCs. Moreover, Sp5 is able to convert mouse epiblast stem cells into a naïve pluripotent state. Thus, Sp5 is an important component of the regulatory network governing mESC naïve pluripotency.

## Abstract (precis)

**Summary:** This study reveals a new function of Sp5 in mouse embryonic stem cell (ESC) self-renewal mediated by CHIR99021 and LIF, and reprogramming of EpiSCs into naїve ESCs.

## INTRODUCTION

Mouse embryonic stem cells (mESCs) are pluripotent cell lines derived from pre-implantation embryos (Evans and Kaufman, 1981; Martin, 1981). They can be expanded in serum-containing medium with LIF, or serum-free N2B27 medium with 2i (hereafter N2B27/2i, see below) *in vitro* while retaining the capacity for multilineage differentiation (Smith, 2001; Williams et al., 1988; Ying et al., 2008). LIF binds to the LIF-receptor–gp130 heterodimer (gp130 is also known as IL6ST), leading to phosphorylation of Stat3 by JAK. Activated Stat3 then translocates into the nucleus and binds DNA (Niwa et al., 1998). Stat3 plays a key role in LIF-mediated mESC self-renewal. A chimeric Stat3 protein fused with the ligand-binding domain of the estrogen receptor (Stat3ER) maintains mESCs in an undifferentiated state in the presence of the synthetic ligand 4-hydroxytamoxifen (4-HT or 4-OHT) without LIF (Matsuda et al., 1999). By contrast, overexpressing a dominant-negative mutant of Stat3 in mESCs blocks LIF-induced activation of endogenous Stat3 and causes differentiation (Niwa et al., 1998). Furthermore, LIF fails to maintain *Stat3*-null mESC self-renewal (Ying et al., 2008). Many Stat3 targets have been identified recently, such as *Klf4*, *Gbx2*, *Pim1*, *Pim3*, *Pramel7*, *Myc* and *Tfcp2l1* (Cartwright et al., 2005; Casanova et al., 2011; Hall et al., 2009; Martello et al., 2013; Tai and Ying, 2013; Ye et al., 2013). When overexpressed, these genes can bypass the LIF requirement for mESC maintenance. However, knockdown of any single gene does not abolish the self-renewal-promoting effect of LIF–Stat3 signaling, suggesting that LIF–Stat3 signaling triggers multiple downstream targets to promote mESC self-renewal.

2i contains two small molecules: CHIR99021 (CHIR) and PD0325901 (PD03), which inhibit glycogen synthase kinase 3 (GSK3) and mitogen-activated protein kinase (MAPK) kinases (MEK proteins), respectively (Huang et al., 2015; Ye et al., 2014; Ying et al., 2008). Inhibiting GSK3 results in the activation of the Wnt/β-catenin signaling pathway. Once in the nucleus, β-catenin liberates many Tcf3-repressed pluripotency genes (Wray et al., 2011), such as *Esrrb*, *Nr0b1*, *Tfcp2l1* and *Nanog* (Martello et al., 2012). It is noteworthy that 2i can sustain *Stat3*-null mESC self-renewal (Ying et al., 2008), whereas the identity of β-catenin-null mESCs can be maintained in medium containing LIF and PD03 (Lyashenko et al., 2011). Taken together, these results suggest CHIR and LIF might share common or cross-compensatory downstream targets in promoting mESC self-renewal. Here, we identified Sp5 as one of common targets of CHIR and LIF, and revealed its new function in promoting mESC self-renewal and reprogramming.

## RESULTS AND DISCUSSION

### Identification of downstream targets of CHIR and LIF in mESCs

**Fig. 1.**
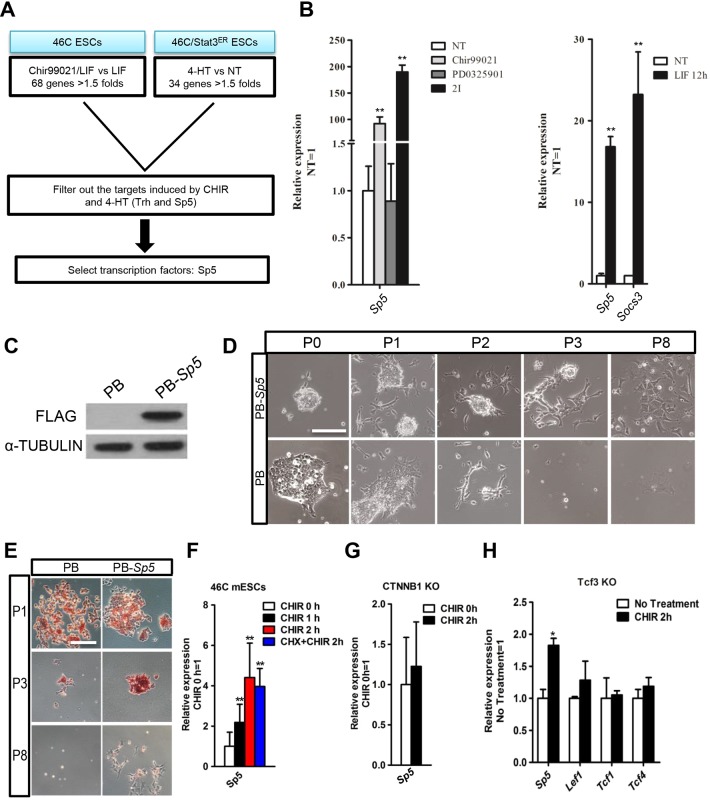
**Overexpressing *Sp5* partially mimics the effect of CHIR in medium with 2i.** (A) Flow chart showing the method used to identify candidate genes downstream of CHIR and Stat3. (B) qRT-PCR analysis of *Socs3* and *Sp5* expression induced by CHIR, PD03 and 2i (left), or LIF (right) for 12 h. Data represent mean±s.d. of three biological replicates. **P<0.01 versus NT. NT, no treatment. (C) FLAG-tagged *Sp5* was introduced into 46C mESCs and the protein level of FLAG-tagged SP5 was determined by western blotting. α-tubulin is used as a loading control. (D,E) Alkaline phosphatase staining images of PB-*Sp5* mESCs cultured in N2B27 medium with PD03 for the indicated number of passages (P). Scale bars: 100 μm. (F–H) qRT-PCR analysis of the indicated gene expression after treatment with CHIR for the indicated time in 46C mESCs, *CTNNB1*-KO (β-catenin knockout) and *Tcf3*-KO mESCs. CHX, cycloheximide treatment. Data represent mean±s.d. of three biological replicates. **P<0.01 versus 0 h CHIR.

To screen the possible shared targets of the Wnt/β-catenin and LIF–Stat3 signaling pathways, we performed a DNA microarray analysis in mESCs treated with or without CHIR (GEO Number: GSE50393). We then looked for genes that were upregulated by 1.5 times or greater by CHIR treatment or by Stat3 stimulation (Bourillot et al., 2009). From this comparison, two common targets emerged: *Trh* and *Sp5* (Fig. 1A). Trh, or thyrotropin-releasing hormone, is a secretory protein. Sp5 (trans-acting transcription factor 5) belongs to the Sp1 transcription factor family (el-Baradi and Pieler, 1991; Wimmer et al., 1993) and harbors three zinc fingers that are similar to those found in members of the KLF gene family (Suske et al., 2005; Treichel et al., 2001). KLF family members, such as Klf2, Klf4 and Klf5, play an important role in maintaining mESC self-renewal (Ema et al., 2008; Hall et al., 2009; Jiang et al., 2008; Qiu et al., 2015). We thus chose *Sp5* as our candidate gene. Quantitative real-time PCR (qRT-PCR) was used to confirm that both CHIR and LIF treatments could induce the expression of *Sp5* (Fig. 1B). *Socs3*, a direct target of LIF–Stat3 signaling, was used as a positive control and showed substantial induction upon LIF stimulation (Fig. 1B) (Niwa et al., 2009).

### Overexpression of *Sp5* promotes mESC self-renewal in the absence of CHIR

We then investigated the function of Sp5 in CHIR- and LIF-mediated self-renewal in mESCs. We generated an mESC line that overexpressed FLAG-tagged *Sp5* using a PiggyBac vector (PB-*Sp5*) in which *Sp5* expression was efficiently enhanced (Fig. 1C). Empty vector (PB) and PB-*Sp5* mESCs grew robustly in N2B27/2i, and in serum-containing medium with LIF. We then withdrew CHIR to test the ability of Sp5 to replace the function of CHIR in N2B27/2i. Before passaging, PB-*Sp5* mESCs maintained an undifferentiated state, whereas cells containing only PB began to die (Fig. 1D,E). Moreover, PB-*Sp5* mESCs could be split for three more passages, but then also collapsed and lost alkaline phosphatase activity (Fig. 1D,E). Therefore, overexpressing *Sp5* supports short-term mESC self-renewal when combined with PD03, indicating that Sp5 partially reproduces the effect of CHIR in 2i.

Sp5 is known to be a downstream target of the Wnt/β-catenin signaling pathway during vertebrate development and in cancer cells (Hoverter et al., 2012; Waaler et al., 2011; Weidinger et al., 2005). To determine whether *Sp5* is regulated through the Wnt/β-catenin signaling pathway in mESCs, we examined the expression of *Sp5* in 46C ESCs after CHIR treatment for 2 h. *Sp5* exhibited signiﬁcantly increased expression in response to CHIR, even in the presence of the protein synthesis inhibitor cycloheximide (Fig. 1F). Furthermore, CHIR failed to induce *Sp5* expression in β-catenin-null ESCs (Fig. 1G), indicating that *Sp5* is a direct target of β-catenin in mESCs. Notably, CHIR upregulated *Sp5* in the absence of *Tcf3* without a change in the expression of other members of the Tcf family, including *Lef1*, *Tcf1* and *Tcf4* (Fig. 1H), indicating that β-catenin can induce *Sp5* expression through interaction with Tcf factors but not Tcf3.

### Overexpression of *Sp5* maintains mESC self-renewal in the absence of LIF

**Fig. 2.**
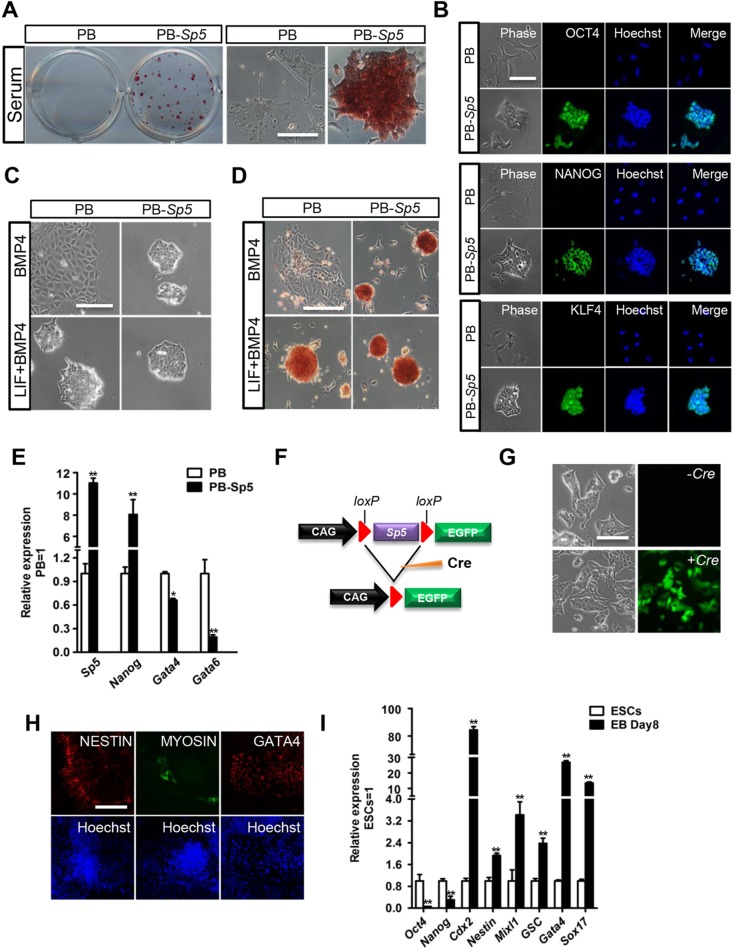
**Forced *Sp5* expression promotes mESC self-renewal without LIF addition.** (A) Alkaline phosphatase staining of PB and PB-*Sp5* mESCs cultured in serum for five passages. Scale bar: 100 μm. (B) Immunofluorescence staining of PB and PB-*Sp5* mESCs cultured in medium with serum for five passages. Scale bar: 100 μm. Hoechst, Hoechst 33342. (C,D) Phase contrast (C) and alkaline phosphatase staining (D) images of PB and PB-*Sp5* mESCs cultured in N2B27 medium with BMP4 in the absence or presence of LIF for 8 days. Scale bars: 100 μm. (E) qRT-PCR analysis of *Sp5*, *Nanog*, *Gata4* and *Gata6* expression in PB and PB-*Sp5* 46C ESCs cultured in medium with LIF and serum. Data represent mean±s.d. of three biological replicates. *P<0.05, **P<0.01 versus PB. (F) Diagram showing the Cre-excisable construct used for *Sp5* overexpression. (G) Phase contrast and GFP images of loxP-*Sp5* mESCs after transfection with or without the *Cre* expression plasmid. ESCs were cultured in medium with LIF and serum. Scale bar: 100 μm. (H) Immunofluorescence staining for the neural marker nestin, the cardiomyocyte marker myosin, and the primitive endoderm marker GATA4 in differentiated cells derived from loxP-*Sp5* mESCs in which the *Sp5* transgene has been removed by Cre. Hoechst 33342 was used for nuclear staining. Scale bar: 100 μm. (I) qRT-PCR analysis of the indicated gene expression in ESCs and embryoid bodies (EB) derived from cells with an excised *Sp5* transgene. Pluripotency genes, *Oct4* and *Nanog*; trophoblast marker, *Cdx2*; ectoderm marker, nestin; mesoderm markers, *Mixl1* and *Gsc*; endoderm makers, *Gata4* and *Sox17*. *P<0.05, **P<0.01 versus ESCs.

To determine the role of Sp5 in LIF–Stat3 signaling, we performed experiments in serum-containing medium without LIF. Surprisingly, PB-*Sp5* mESCs could be continually passaged while retaining typical ESC morphology, positive alkaline phosphatase activity and high expression levels of the pluripotency markers OCT4, NANOG and KLF4, whereas empty vector (PB) control cells differentiated (Fig. 2A,B; Fig. S1A–C). The results were validated in OCRG9 mESCs harboring an Oct4–GFP pluripotency reporter (Fig. S1D,E). We then tried culturing PB and PB-*Sp*5 46C ESCs in N2B27 medium with BMP4, which maintains mESC self-renewal when combined with LIF (Ying et al., 2003). Similarly, PB-*Sp5* mESCs maintained an undifferentiated state, whereas PB cells differentiated rapidly (Fig. 2C,D). We found that overexpression of *Sp5* led to an upregulation in the level of *Nanog*, whereas it repressed the expression of the differentiation markers *Gata4* and *Gata6*. Both of these markers are typically suppressed by *Nanog* in mESCs (Fig. 2E) (Chambers et al., 2003). These collective results suggest that elevated expression of *Sp5* recapitulates the self-renewal-promoting effect of the LIF–Stat3 signaling pathway, probably through induction of *Nanog* expression.

To examine whether mESCs maintained by *Sp5* overexpression retain pluripotency, we used a *loxP*-based excisable vector to overexpress *Sp5*. After expansion in the absence of LIF for five passages, the ﬂoxed-*Sp5* mESCs were transiently transfected with a *Cre* expression vector to excise the floxed *Sp5* transgene (Fig. 2F). After the excision of *Sp5* transgene, the *GFP* transgene was activated, and their maintenance became dependent on LIF (Fig. 2G). The revertant cells maintained the ability to differentiate into cells from the three germ layers after forming embryoid bodies (Fig. 2H,I), suggesting that Sp5 overexpressing ESCs retain their pluripotency.

### Overexpressing *Sp5* sustains *Stat3*-null ESCs in an undifferentiated state

To determine whether Stat3 is dispensable for *Sp5*-promoted mESC self-renewal, we overexpressed *Sp5* in *Stat3*-null mESCs and cultured them in serum-containing medium without LIF. These transfectants remained undifferentiated, whereas *Stat3*-null mESCs transfected with an empty vector died or differentiated after two passages (Fig. S2). These results suggest that the self-renewal-promoting effect of Sp5 is independent of Stat3.

### *Sp5* is a direct downstream target of the LIF–Stat3 signaling pathway

**Fig. 3.**
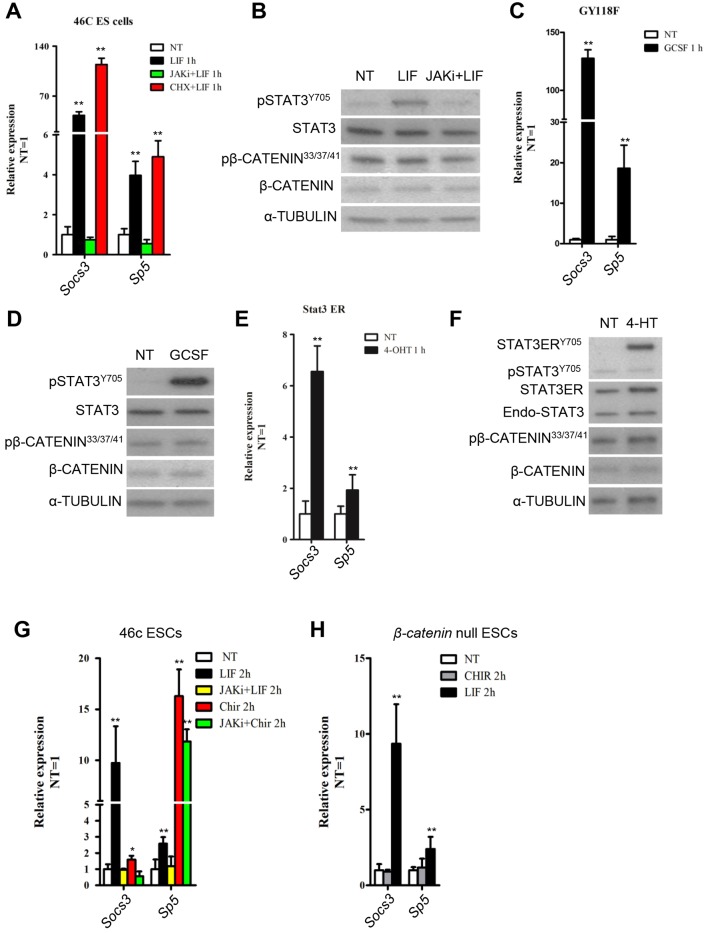
***Sp5* is a direct target of Stat3.** (A) qRT-PCR analysis of *Socs3* and *Sp5* expression levels in 46C mESCs deprived of LIF overnight and stimulated with LIF for 1 h in the presence or absence of JAK inhibitor I (JAKi) or cycloheximide (CHX, 50 mg/ml). Data represent mean±s.d. of three biological replicates. **P<0.01 vesus 46C NT. NT, no treatment. (B,C) qRT-PCR analysis of *Socs3* and *Sp5* in GY118F and Stat3ER transfectants deprived of LIF overnight and stimulated with GCSF or 4-HT for 1 h. Data represent mean±s.d. of three biological replicates. **P<0.01 versus NT. (D–F) Western blot analysis of total and phosphorylated Stat3 and β-catenin in wild type, and GY118F- and Stat3ER-transfected mESCs. p, phosphorylated protein. (G,H) qRT-PCR analysis of *Socs3* and *Sp5* in β-catenin-null or 46C mESCs treated with LIF or CHIR for 2 h in the presence or absence of JAK inhibitor I. Data represent mean±s.d. of three biological replicates. *P<0.05, **P<0.01 versus NT.

LIF mainly activates three intracellular signaling pathways: the JAK–Stat3, PI3K–AKT and MAPK pathways (Hirai et al., 2011). To examine whether Sp5 is a direct target of LIF–Stat3 pathway, we tested the responsiveness of *Sp5* to the activation of Stat3. LIF stimulation for 1 h in 46C mESCs led to an accumulation of phosphorylated STAT3 and acute transcript induction of *Socs3* and *Sp5* (Fig. 3A,B). However, their expression was suppressed by JAK inhibitor (Fig. 3A). This phenomenon was also observed in the presence of the protein synthesis inhibitor cycloheximide (Fig. 3A,B). A similar expression pattern of *Sp5* induction was observed in GY118F and Stat3ER transfectants (Fig. 3C–F). GY118F is a mutated chimeric receptor and only triggers JAK–Stat3 signaling in the presence of granulocyte colony stimulating factor (GCSF) (Burdon et al., 1999). Stat3ER stimulates Stat3 direct targets by translocating into the nucleus in the presence of 4-HT. This process occurs independently of endogenous STAT3, MAPK and AKT (Bourillot et al., 2009; Matsuda et al., 1999). Collectively, these data indicate that Sp5 is a direct target of the LIF–Stat3 signaling pathway. However, knockdown of *Sp5* did not impair the self-renewal-promoting effect of LIF–Stat3 signaling (Fig. S3A,B). Interestingly, downregulation of *Sp5* significantly induced *Pim1* (a Stat3 direct target) expression (Fig. S3A) (Martello et al., 2013), suggesting that the self-renewal-promoting function of Sp5 is redundant with other Stat3 targets in mESCs.

### LIF and CHIR upregulates *Sp5* expression independently

Given that Sp5 is also a direct target of the Wnt/β-catenin signaling pathway (Dunty et al., 2014; Weidinger et al., 2005), we next wanted to examine whether LIF and CHIR depended on each other to exert their own upregulating effect. CHIR strongly induced *Sp5* expression in 46C mESCs in the presence of JAK inhibitor whereas LIF failed (Fig. 3G). By contrast, LIF stimulated *Sp5* expression in the absence of β-catenin, whereas CHIR-mediated induction of *Sp5* was dependent on β-catenin (Fig. 3H), indicating that LIF and CHIR induce *Sp5* transcription independently of one another.

### *Sp5* is able to reprogram EpiSCs to a naïve state

**Fig. 4.**
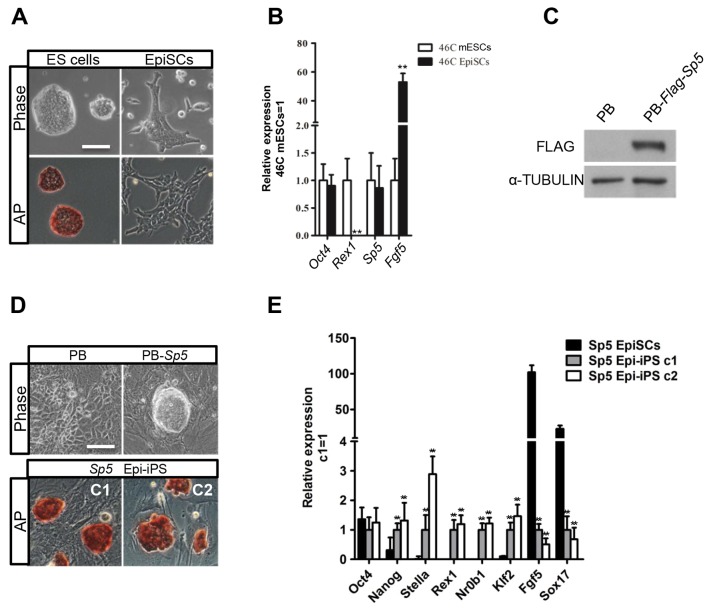
**Forced expression of *Sp5* promotes EpiSC reprogramming to naïve pluripotency.** (A) Phase contrast and alkaline phosphatase (AP) staining images of 46C mESCs and 46C mESC-derived EpiSCs. Scale bar: 100 μm. (B) qRT-PCR analysis of expression patterns of *Sp5*, *Oct4*, *Rex1* and *Fgf5* in 46C mESCs and EpiSCs. Data represent mean±s.d. of three biological replicates. **P<0.01 versus 46C mESCs. (C) Western blot analysis for Sp5 in 46C EpiSCs transfected with vector expressing FLAG-tagged *Sp5*. (D) Phase contrast and alkaline phosphatase staining images of *Sp5* EpiSC transfectants and *Sp5* EpiSC transfectant-derived induced pluripotent stem cells (iPSCs). Scale bar: 100 μm. (E) qRT-PCR analysis of *Oct4*, *Nanog*, *Stella*, *Nr0b1*, *Klf2*, *Rex1*, *Fgf5* and *Sox17* expression in *Sp5* EpiSCs and two *Sp5* Epi-iPS colonies. Data represent mean±s.d. of three biological replicates. **P<0.01 versus *Sp5* EpiSCs. C1 and C2 are the individual colonies of ESC-like cell. Epi-iPS, EpiSC-derived iPSCs.

Another distinguishing feature of LIF–Stat3 signaling is its ability to convert mouse epiblast stem cells (EpiSCs) into cells with a naïve state of pluripotency (van Oosten et al., 2012; Yang et al., 2010). Mouse EpiSCs are isolated from epiblasts of post-implantation mouse embryos and share many features with human ESCs (Brons et al., 2007; Tesar et al., 2007). *Stat3* and its multiple targets are expressed highly in mESCs, but are downregulated in EpiSCs. Importantly, their overexpression is sufficient to reprogram EpiSC into the mESC state (Guo et al., 2009; Tai and Ying, 2013; Yang et al., 2010). To test the role of Sp5 in EpiSC reprogramming, we first differentiated 46C mESCs into EpiSCs (Fig. 4A), but observed no obvious change in *Sp5* expression (Fig. 4B). We then engineered 46C EpiSCs to overexpress *Sp5* (Fig. 4C) and cultured 10^6^ transfectants in medium containing LIF, serum and 2i. After 12 days, five ESC-like colonies emerged, whereas empty vector transfectants died or differentiated (Fig. 4D). We then picked two colonies and these colonies continuously expanded in medium containing serum and LIF. They expressed high levels of the naïve pluripotency markers *Nanog*, *Stella*, *Nrob1*, *Klf2* and *Rex1*, but low levels of the EpiSC-specific markers *Fgf5* and *Sox17* (Fig. 4E). We confirmed this experiment in E3 EpiSCs carrying an Oct4–GFP pluripotency reporter (Greber et al., 2010). The *Sp5*-reprogrammed stem cells were expanded in medium containing serum and LIF over multiple passages, showing undifferentiated morphology and stable expression of the Oct4–GFP reporter (Fig. S4A,B). These results imply that forced expression of *Sp5* is capable of converting EpiSCs into a naïve pluripotent state.

### Conclusions

CHIR and LIF have redundant function in mESC maintenance. We demonstrate that Sp5 is a convergent target of the Wnt/β-catenin and LIF–Stat3 signaling pathways. In support of this, we present evidence that *Sp5* recapitulates features of Wnt/β-catenin and LIF–Stat3 signaling in promoting mESC self-renewal. Furthermore, overexpression of *Sp5* is able to overcome the differentiation cues to convert EpiSCs into cells with naïve pluripotency. Our study therefore reveals a new function of Sp5 in mESC self-renewal, which is mediated by CHIR and LIF, and reprogramming of EpiSCs into naïve ESCs. In the future, understanding how Sp5 cooperates with other downstream targets of β-catenin and Stat3 to maintain the pluripotent ESC state might facilitate the development of new culture conditions for the derivation of authentic ESCs from various species.

## MATERIALS AND METHODS

### Cell culture

46C mESCs, kindly provided by Austin Smith (Wellcome Trust-Medical Research Council Cambridge Stem Cell Institute, University of Cambridge, UK), were cultured on 0.1% gelatin-coated dishes. mESC medium contains Glasgow's minimum essential medium (GMEM; Sigma), 10% fetal calf serum (FCS; HyClone), 1% MEM nonessential amino acids (Invitrogen), 2 mM GlutaMax (Invitrogen), 0.1 mM β-mercaptoethanol (Invitrogen) and 100 units/ml LIF (prepared in-house). For serum-free culture, mESCs were maintained in N2B27 supplemented with 3 µM CHIR99021 and 1 µM PD0325901 (Sigma), or supplemented with 100 units/ml LIF and 10 ng/ml BMP4 (Peprotech).

### Plasmid construction and cell transfection

The coding region of *Sp5* was inserted into a PiggyBac vector and transduced into cells combined with 2 µg transposase using LTX (Invitrogen). Selection was continued for 1 week by adding 2 µg/ml puromycin. The short hairpin RNA (shRNA) plasmids were generated according to the Addgene PLKO.1 protocol. The target-specific sequence for *Sp5* is 5′-GGATTCAAAGGATTTGCTTTC-3′.

### Alkaline phosphatase activity assay and qRT-PCR

The alkaline phosphatase activity assay and qRT-PCR analyses were performed according to our previous report (Ye et al., 2013). The primers used for the qRT-PCR analyses are listed in Table S1.

### Western blotting

Western blotting was performed according to a standard protocol. The primary antibodies used for probing were against α-tubulin (32-2500, Invitrogen; 1:2000), phosphorylated Stat3^Y705^ (9131S, Cell Signaling Technology; 1:1000), Stat3 (610190, BD Biosciences; 1:1000), phosphorylated β-Catenin^Ser33/Ser37/Thr41^ (9561S, Cell Signaling Technology; 1:1000) and β-catenin (610153, BD Biosciences; 1:1000).

### Immunofluorescence staining

Immunostaining was performed with standard protocols. Primary antibodies used were against Oct4 (sc-5279, Santa Cruz Biotechnology; 1:200), Nanog (AF2729, R&D; 1:100), Klf4 (AF3158, R&D; 1:100), nestin (2Q178, Santa Cruz Biotechnology, 1:100), GATA4 (G4, Santa Cruz Biotechnology, 1:100) and myosin (MF-20, Developmental Studies Hybridoma Bank, 1:50).

### mEpiSC derivation and reprogramming

For ESC-to-EpiSC differentiation, 5000 46C mESCs were plated into 0.1% gelatin-coated plates and cultured in serum medium supplemented with activin A (10 ng/ml, Peprotech), bFGF (10 ng/ml, Peprotech) and JW55 (20 µm, Tocris). Cells were used after 10 passages. For reprogramming, 10^6^ transfectants were seeded onto a 3.5-cm dish and cultured in mESC medium supplemented with LIF and 2i. The number of alkaline-phosphatase-positive clones was counted under a microscope. ESC-like clones were picked and subsequently expanded in medium containing LIF and serum.

### Accession numbers

Data and details of the method for the DNA microarray analysis are available in the Gene Expression Omnibus under accession number GSE50393.

### Statistical analysis

All data are reported as the mean±s.d. A Student's *t*-test was used to determine the significance of differences in comparisons. Values of *P*<0.05 were considered as statistically significant.
